# Aberrant activity of NKL homeobox gene NKX3-2 in a T-ALL subset

**DOI:** 10.1371/journal.pone.0197194

**Published:** 2018-05-10

**Authors:** Stefan Nagel, Corinna Meyer, Maren Kaufmann, Margarete Zaborski, Roderick A. F. MacLeod, Hans G. Drexler

**Affiliations:** Department of Human and Animal Cell Lines, Leibniz-Institute DSMZ—German Collection of Microorganisms and Cell Cultures, Braunschweig, Germany; German Cancer Research Center (DKFZ), GERMANY

## Abstract

T-cell acute lymphoblastic leukemia (T-ALL) is a hematopoietic malignancy originating from T-cell progenitors in which differentiation is blocked at early stages. Physiological expression of specific NKL homeobox genes obeys a hematopoietic NKL-code implicated in the process of lymphopoiesis while in differentiated T-cells these genes are silenced. We propose that this developmental expression pattern underlies the observation that NKL homeobox genes are the most ubiquitous group of transcription factors deregulated in T-ALL, including TLX1, TLX3, NKX2-5 and NKX3-1. Here, we describe a novel member of the NKL homeobox gene subclass, NKX3-2 (BAPX1), which is aberrantly activated in 18% of pediatric T-ALL patients analyzed while being normally expressed in developing spleen. Identification of NKX3-2 expression in T-ALL cell line CCRF-CEM qualified these cells to model its deregulation and function in a leukemic context. Genomic and chromosomal analyses demonstrated normal configuration of the NKX3-2 locus at chromosome 4p15, thus excluding cytogenetic dysregulation. Comparative expression profiling analysis of NKX3-2 patient data revealed deregulated activity of BMP- and MAPK-signalling. These candidate pathways were experimentally confirmed to mediate aberrant NKX3-2 expression. We also show that homeobox gene SIX6, plus MIR17HG and GATA3 are downstream targets of NKX3-2 and plausibly contribute to the pathogenesis of this malignancy by suppressing T-cell differentiation. Finally, NKL homeobox gene NKX2-5 was activated by NKX3-2 in CCRF-CEM and by FOXG1 in PEER, representing mutually inhibitory activators of this translocated oncogene. Together, our findings reveal a novel oncogenic NKL homeobox gene subclass member which is aberrantly expressed in a large subset of T-ALL patients and participates in a deregulated gene network likely to arise in developing spleen.

## Introduction

T-cell acute lymphoblastic leukemia (T-ALL) is an hematopoietic cancer affecting the lymphoid lineage. It is a rare malignancy and represents about 15% of childhood and 25% of adult ALL. However, T-ALL patients have a poor prognosis. Therefore, this disease deserves reinforced investigation and novel therapies. Normal T-cell differentiation is basically regulated at the transcriptional level [1,2]. Accordingly, several types of oncogenes in T-ALL encode transcription factors (TF) whose deregulation contributes to cell transformation and differentiation arrest at particular stages in T-cell progenitors [3,4]. In many cases chromosomal aberrations mediate their deregulated activity [4]. Such as oncogene TAL1 which is aberrantly activated via a small upstream microdeletion at chromosome 1p13 generating the fusion gene STIL-TAL1, or via mutational generation of a super-enhancer in its regulatory region [5,6]. This gene is a member of the basic helix-loop-helix (bHLH) family of TF and normally exhibits transcriptional activity restricted to the early stages of hematopoiesis. Oncogene NKX2-5 encodes a homeodomain containing TF and is activated via chromosomal translocation t(5;14)(q35;q32) [7]. This rearrangement juxtaposes far upstream enhancers of the T-cell regulator gene BCL11B with flanking regions of the NKX2-5 gene which is normally silenced in hematopoietic cells [8].

Homeobox genes are implicated in basic developmental processes during embryogenesis and in the adult [9]. Sequence differences affecting their conserved homeodomain have been used to (sub)classify this prominent group of TFs [10]. NKL and SIX represent two subclasses of the ANTP and SINE class, respectively, encompassing several members deregulated in leukemia and lymphoma [11–13]. Physiologically, NKL homeobox genes act in developmental processes of particular tissues and organs like NKX2-5 in heart, thymus and spleen, TLX1 in dorsal root ganglia and spleen, and NKX3-1 in the prostate [14–16]. Reportedly, more than 20 NKL homeobox genes are aberrantly activated in T-ALL [17,18]. Nine physiologically expressed members of this subclass constitute an NKL-code in early hematopoiesis and lymphopoiesis [17]. The importance of this basic developmental gene pattern may underlie the high frequency and thus the predisposition for aberrant activities of these TFs in hematopoietic malignancies, notably T-ALL. Human T-ALL cell lines expressing particular oncogenic NKL homeobox genes represent useful bench models to investigate their biological role(s) in this malignancy. Hitherto, model T-ALL cell lines have been described for TLX1 (ALL-SIL), TLX3 (HPB-ALL, DND-41), NKX2-5 (CCRF-CEM, PEER), NKX3-1 (HSB-2, JURKAT, MOLT-14, PER-117, PF-382, RPMI-8402), and MSX1 (LOUCY, PER-117) [7,19–22].

Aberrant activity of NKL homeobox gene NKX3-1 has been detected in T-ALL patients, mostly coexpressing bHLH oncogene TAL1 and SIX subclass member SIX6 [12]. Thus, the TF complex comprising TAL1, GATA3 and LMO is a direct activator of NKX3-1 while SIX6 is in turn a direct target of NKX3-1 [21,23]. Homeobox gene SIX6 encodes a differentiation factor normally controlling ocular development and might thus promote NKL-mediated oncogenic activity by deregulating differentiation processes [24]. Here, we identify NKX3-2 as an additional direct activator of SIX6 and characterize the expression and deregulation of this novel NKL homeobox gene in T-ALL.

## Materials and methods

### Cell lines and treatments

Authenticated cell lines drawn from the DSMZ Cell Lines Bank (Braunschweig, Germany) were cultivated as described previously [25]. Cell stimulations were performed by treatment with recombinant human bone morphogenetic protein (BMP)4, fibroblast growth factor (FGF)2, transforming growth factor beta (TGFb) and tumor necrosis factor alpha (TNFa) for 16h at concentrations of 20 ng/ml (R&D Systems, Wiesbaden, Germany). Treatments with BMP receptor inhibitor dorsomorphin (DM) obtained from Calbiochem (Darmstadt, Germany) and dissolved in dimethyl sulfoxide (DMSO) were performed for 16h at 5 μM. MAPK-inhibitor PD98059 was obtained from Sigma (Taufkirchen, Germany) and used at 25 μM for 16h. To suppress NFkB-signalling we used NFkB Activation Inhibitor (Calbiochem) at 14 μM for 16 h.

Using the EPI-2500 impulse generator (Fischer, Heidelberg, Germany) at 350 V for 10 ms, 2 μg expression constructs or 80 pmol siRNA were transfected into the cells. After incubation for 16 h the cells were harvested. Expression constructs (NKX3-1, NKX3-2 and FOXG1) were obtained from Origene (Wiesbaden, Germany). SiRNA oligonucleotides and AllStars negative control siRNA (termed here siCTR) were obtained from Qiagen (Hilden, Germany).

### Expression profiling

All expression profiling data analysed in this study were generated from gene chips HG U133 Plus 2.0 (Affymetrix, High Wycombe, UK). For analysis of primary human cells we used Gene Expression Omnibus dataset GSE42038 which comprises 79 pediatric T-ALL patient samples. Analyses of these expression data were performed using R-based online tools which compute 250 coexpressed genes and their statistical significance. Public R-software was used for further statistical calculations and graphical visualization (http://www.bioconductor.org/).

T-ALL cell line datasets were generated by Prof. Andreas Rosenwald (Institute of Pathology, University of Würzburg, Germany) and by Dr. Robert Geffers (Genome Analytics Facility, Helmholtz Centre for Infection Research, Braunschweig, Germany) and are available via NCBI/GEO dataset GSE87334. For analyses of differential gene activities data were transformed as follows: after RMA-background correction and quantile normalization of the spot intensities, profiling data were expressed as ratios of the sample mean and subsequently log2 transformed. Data processing was performed via R/Bioconductor using Limma and Affy packages. For creation of heat maps and clustering of selected genes we used CLUSTER version 3.0 and TREEVIEW version 1.60 (http://rana.lbl.gov/EisenSoftware.htm).

### Chromosomal and genomic analyses

Spectral karyotyping (SKY) and fluorescent in-situ hybridization (FISH) were performed as described previously [26]. SKY-probes were obtained from Applied Spectral Imaging (Neckarhausen, Germany). For FISH we used RP11-BAC clones from BacPac Resources, Children´s Hospital Oakland Research Institute (CA, USA). Insert DNA was harvested using the Big BAC DNA Kit (Princeton Separations, Adelphia, NJ, USA) and directly labelled by nick translation with dUTP-fluors (Dyomics, Jena, Germany). Fluorescent photographs were taken with an Axio-Imager microscope (Zeiss, Göttingen, Germany). The images were analyzed using a dual Spectral Imaging FISH system (Applied Spectral Imaging).

Dr. Robert Geffers (Helmholtz Centre for Infection Research) performed the genomic profiling using CytoScan HD Arrays (Affymetrix). They enable the detection of high resolution copy number across the genome. These high density array contain more than 2.4 million markers for copy number and about 750.000 genotype-able SNPs to perform high resolution copy number, breakpoint estimation, and detection of loss of heterozygosity. The 1.7 million unique non-polymorphic probes cover RefSeq genes, OMIM genes, and cytogenetic relevant regions. The genomic DNA of the cell lines was prepared using the Qiagen Gentra Puregene Kit (Qiagen). The performance including labelling, hybridization and washing was done using kits, CytoScan HD arrays and protocols according to the manufacturer (Affymetrix). Data analyses were performed using the Chromosome Analysis Suite software version 3.1.0.15 (Affymetrix).

### Polymerase Chain Reaction (PCR) analyses

Using TRIzol reagent (Invitrogen, Karlsruhe, Germany) we extracted total RNA from cell lines. Total RNA from primary human cells and tissues was commercially obtained. We used peripheral mononuclear blood cells (PMBC), CD3-positive T-cells and CD19-positive B-cells (Miltenyi Biotec, Bergisch Gladbach, Germany), retina, spleen and thymus (Biochain, Newark, CA, USA). We used 5 μg RNA and Superscript II (Invitrogen) to synthesize cDNA by random priming.

Quantification of gene transcripts was performed by Real-Time quantitative polymerase chain reaction (RQ-PCR) using the 7500 Fast Real-Time System and commercial primer sets (Applied Biosystems, Darmstadt, Germany). We used TATA box binding protein (TBP) for normalization of expression levels. Quantitative analyses were performed in triplicate. The standard deviations in the figures were presented as error bars. The statistical significance was calculated by an R-based T-Test. The obtained p-values were indicated by asterisks (* p<0.05, ** p<0.01, *** p<0.001, n.s. no significance).

### Protein analyses

Western blots were generated by the semi-dry method. Protein lysates from cell lines were prepared using SIGMAFast protease inhibitor cocktail (Sigma). Cell lysates from primary human spleen were obtained from Origene. Proteins were transferred onto nitrocellulose membranes (Bio-Rad, München, Germany) and blocked with 5% dry milk powder dissolved in phosphate-buffered-saline buffer (PBS). The following antibodies were used: alpha-Tubulin (Sigma), ERK and phospho-ERK (Santa Cruz Biotechnology, Heidelberg, Germany) and FOXG1 (Thermo Fisher, Rockford, IL, USA). For loading control blots were reversibly stained with Poinceau (Sigma) and detection of alpha-Tubulin (TUBA) was performed thereafter. Secondary antibodies were linked to peroxidase for detection by Western-Lightning-ECL (Perkin Elmer, Waltham, MA, USA). Documentation was performed using the digital system ChemoStar Imager (INTAS, Göttingen, Germany).

Immuno-cytology was performed as follows: cells were spun onto slides and subsequently air-dried and fixed with methanol/acetic acid for 90 s. NKX3-2 antibody (Thermo Fisher) was diluted 1:20 in PBS containing 5% BSA, incubated for 30 min. Washing was performed 3 times with PBS. Preparations were incubated with secondary antibody (diluted 1:100) for 20 min. After final washing the cells were mounted in Vectashield (Vector Laboratories, Burlingame, CA), containing DAPI for nuclear staining. Documentation of subcellular protein localization was performed using an Axio-Imager microscope (Zeiss, Göttingen, Germany) configured to a dual Spectral Imaging FISH system (Applied Spectral Imaging).

### Reporter gene analysis

The reporter gene construct was synthesized combining a reporter gene with a genomic fragment derived from the intragenic region of SIX6 which contains a binding site for NKX3-1 as described previously [21]. RQ-PCR using a commercial HOXA9 assay quantified the spliced reporter-transcript which corresponds to the promoter activity.

### Cell cycle analysis

The cells were treated for SIX6 knockdown using siRNAs as described above. After 48h the cells were fixed with 70% ethanol (-20°C) and stored on ice, washed with PBS and stained with 20 μg/ml propidium iodide (Sigma). Then, the DNA content of the cells was determined by flow cytometry using FACSCalibur (Becton Dickinson, Heidelberg, Germany).

## Results

### Aberrant expression of NKX3-2 in T-ALL

**Fig 1:**
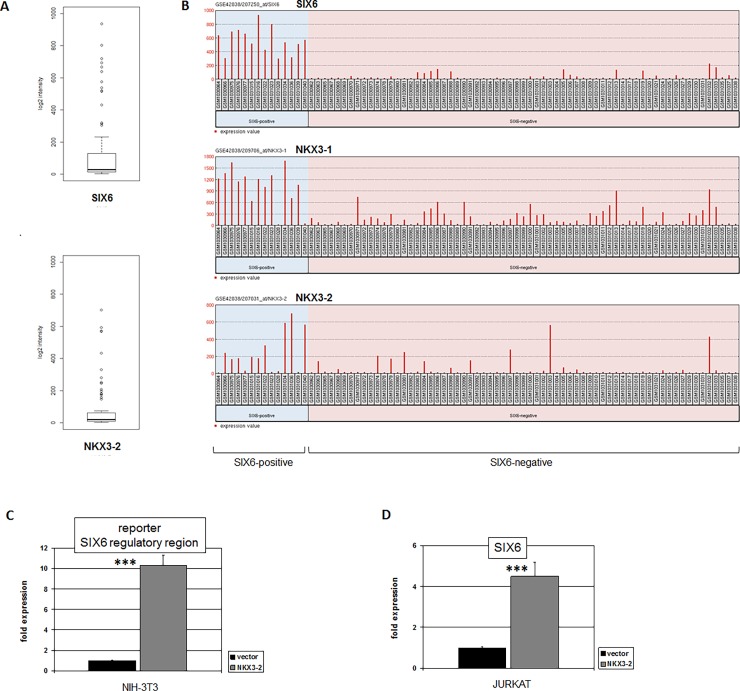
Expression and regulation of SIX6 in T-ALL. (A) Boxplots show expression data from T-ALL dataset GSE42038 for SIX6 (above) and NKX3-2 (below). Patient samples exhibiting gene overexpression are indicated by circles totaling 13/79 (16%) for SIX6 and 14/79 (18%) for NKX3-2. (B) Expression levels of SIX6, NKX3-1 and NKX3-2 for pediatric T-ALL patients obtained from dataset GSE42038 are ranked according SIX6-overexpression and control remainders. (C) Reporter gene assay using a genomic fragment from the SIX6 gene containing a confirmed NKX3-1 binding site demonstrated direct activation of SIX6 by NKX3-2 in NIH-3T3 cells. (D) Forced overexpression of NKX3-2 in JURKAT cells resulted in enhanced expression levels of SIX6 as analyzed by RQ-PCR.

Homeobox gene SIX6 is reportedly coexpressed with NKX3-1 in T-ALL patients and cell lines, the latter used to demonstrate direct transcriptional activation of SIX6 by NKX3-1 [12,21]. However, published T-ALL expression data of both patients and cell lines show SIX6 positive instances which are NKX3-1 negative, implying alternative mechanisms of SIX6 activation. To identify additional SIX6 activators in T-ALL we analyzed public expression profiling dataset GSE42038 containing 79 pediatric T-ALL patient samples. Statistical calculation showed that this dataset includes 13 patient samples which significantly overexpress SIX6 (**Fig 1A**). Bioinformatic comparison of these SIX6-positive cases with the remaining SIX6-negative controls revealed 250 most significantly correlated genes including NKX3-1 (p = 3.1x10^-17^) and NKX3-2 (p = 2.1x10^-6^) (**Fig 1B**). The close relationship of these NKL homeobox genes was taken to indicate that NKX3-2 may activate SIX6 as shown previously for NKX3-1. Therefore, we performed a reporter gene assay using a genomic fragment from the SIX6 gene containing the confirmed NKX3-1 binding site located in exon 2 [21]. This assay demonstrated direct activation of SIX6 by NKX3-2 (**Fig 1C**). Moreover, forced expression of NKX3-2 in the SIX6-positive T-ALL cell line JURKAT resulted in enhanced expression levels of SIX6 transcripts (**Fig 1D**). Thus, both NKX3-1 and NKX3-2 directly activate SIX6 in T-ALL.

**Fig 2:**
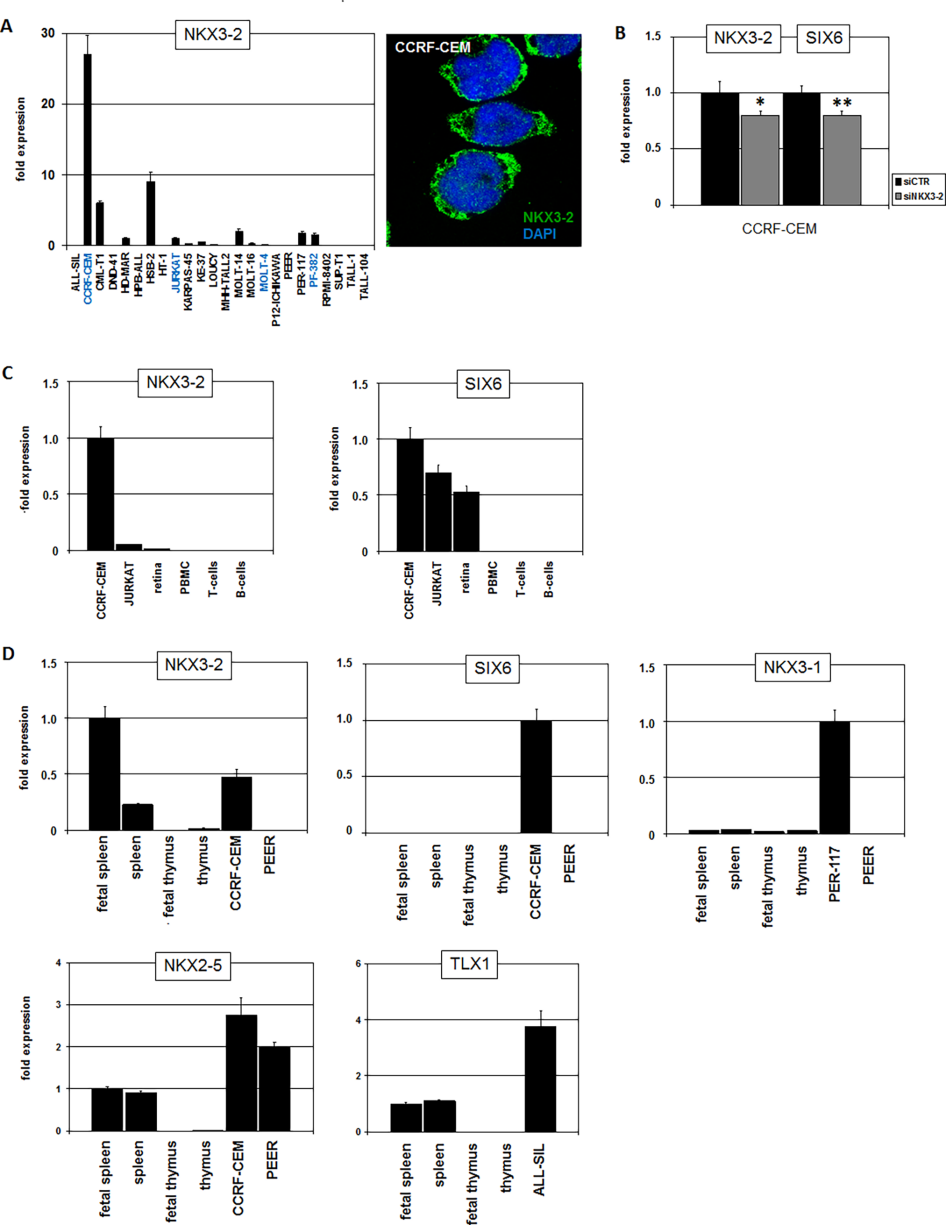
Expression of NKX3-2 and SIX6 in cell lines and primary tissues. (A) RQ-PCR analysis of NKX3-2 expression in 24 T-ALL cell lines demonstrated enhanced expression in CCRF-CEM (left). Values are given relative to JURKAT. SIX6-positive cell lines are indicated in blue [21]. NKX3-2 protein was detected in CCRF-CEM by immuno-cytology (right). (B) SiRNA-mediated knockdown of NKX3-2 in CCRF-CEM resulted in concomitantly reduced expression of NKX3-2 and SIX6, supporting SIX6 activation by NKX3-2. The indicated values were given in relation to the siRNA-control which were set to 1. (C) RQ-PCR analysis of NKX3-2 (left) and SIX6 (right) in T-ALL cell lines and primary tissues including retina and blood cells. (D) RQ-PCR analysis of NKX3-2, SIX6, NKX3-1, NKX2-5 and TLX1 in T-ALL cell lines and primary tissues including spleen and thymus.

Further analysis of dataset GSE42038 showed NKX3-2 overexpression in 14/79 (18%) of the pediatric T-ALL patients (**Fig 1A**), highlighting this factor as a frequently expressed NKL homeobox gene in a large subset of this malignancy deserving further investigation. In search of a model for NKX3-2 we screened 24 T-ALL cell lines by RQ-PCR for its gene activity, finding enhanced NKX3-2 transcript levels in CCRF-CEM (**Fig 2A**). Protein expression examined by immuno-cytology showed NKX3-2 present in nuclear speckles and in the cytoplasm of CCRF-CEM (**Fig 2A**). These subcellular localizations indicated operations as TF in addition to regulation via cytoplasmic protein interaction. Subsequent siRNA-mediated knockdown of NKX3-2 in this cell line resulted in decreased SIX6 levels, confirming the proposed regulatory impact (**Fig 2B**). CCRF-CEM has been previously shown to express SIX6 but not NKX3-1 [21], thus supporting the view that SIX6 is a target of both NKX3-1 and NKX3-2. Next we investigated physiological gene activities of NKX3-2 and SIX6 in primary tissue samples (**Fig 2C**). Considerable SIX6 expression levels were detected in retina cells, confirming the described role of this homeobox gene in eye development [24]. However, NKX3-2 expression was not detected in the retina, indicating absence of a regulatory connection in this tissue. Furthermore, neither SIX6 nor NKX3-2 showed significant expression levels in normal hematopoietic cells including PBMCs, T-cells and B-cells, confirming their aberrant activity in T-ALL subsets.

NKX3-2 has been described as TF playing a role in murine spleen development [27]. Therefore, we quantified expression levels of NKX3-2 and SIX6 in human fetal and adult spleen and additionally in thymus (**Fig 2D**). While SIX6 was undetectable in these tissues, NKX3-2 expression was confirmed in the spleen. The expression level of NKX3-2 was higher in CCRF-CEM as compared to adult spleen but lower than in fetal spleen. These data suggest that in T-cell progenitors NKX3-2 reactivates specific differentiation programs which normally operate during development of the spleen. In contrast, expression of prostate-specific NKX3-1 was neither detected in the spleen nor in the thymus (**Fig 2D**). Additional NKL homeobox genes regulating development of the spleen are NKX2-5 and TLX1 [28]. Both transcripts were detected in spleen samples analyzed and additionally in those T-ALL cell lines which contain activating chromosomal translocations at NKX2-5 (CCRF-CEM, PEER) and TLX1 (ALL-SIL) (**Fig 2D**). Thus, NKX3-2 in addition to TLX1 and NKX2-5 represent oncogenic NKL homeobox genes in T-ALL which are physiologically expressed in the spleen. The high frequency of deregulated NKX3-2 and the shared target gene SIX6 with NKX3-1 may indicate that this homeobox gene is an important pathological factor.

### Activation of NKX3-2 in T-ALL

**Fig 3:**
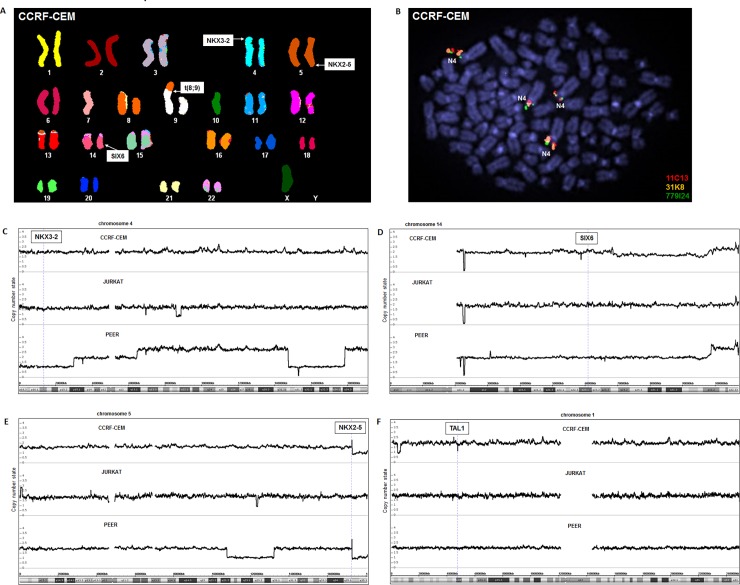
Chromosomal and genomic analyses of NKX3-2. (A) SKY analysis of CCRF-CEM revealed the aberration t(8;9). The loci for NKX3-2, NKX2-5 and SIX6 are indicated. (B) FISH analysis of the NKX3-2 locus in CCRF-CEM demonstrates wild type configurations (N4: normal chromosome 4). Genomic profiling data of T-ALL cell lines CCRF-CEM, JURKAT and PEER for chromosome 4 (C), chromosome 14 (D), chromosome 5 (E), and chromosome 1 (F). The localizations of selected genes including NKX3-2, SIX6, NKX2-5 and TAL1 are indicated.

Several oncogenic NKL homeobox genes in T-ALL are aberrantly activated via chromosomal translocations like t(10;14)(q24;q11) juxtaposing a TCR-enhancer with TLX1 or t(5;14)(q35;q32) a BCL11B-enhancer with TLX3 or NKX2-5 [7,19,20]. To examine the chromosomal configuration of the NKX3-2 locus in CCRF-CEM we performed SKY (**Fig 3A**). This analysis revealed the uncharacterized chromosomal rearrangement t(8;9), but no alterations at the loci of NKX3-2 (4p15) or SIX6 (14q23). Of note, t(5;14)(q35;q32) targeting NKX2-5 in this cell line was not detectable by this method, probably due to the submicroscopic dimensions of the rearranged fragments which lay below those visualized by SKY. To circumvent similar technical limitations we additionally performed FISH using straddling and flanking probes for NKX3-2 (**Fig 3B**), an exercise which also showed wild type configurations at NKX3-2. Finally, to look for small copy number alterations neither detectable by SKY nor by FISH we performed genomic profiling of CCRF-CEM in comparison to JURKAT and PEER. While this assay visualized the known aberrations at NKX2-5 (CCRF-CEM, PEER) and TAL1 (CCRF-CEM), no abnormalities were detected at NKX3-2 and SIX6 in CCRF-CEM, JURKAT or PEER (**Fig 3**). Thus, chromosomal and genomic alterations are unlikely to account for deregulated NKX3-2 expression in T-ALL cell line CCRF-CEM.

**Fig 4:**
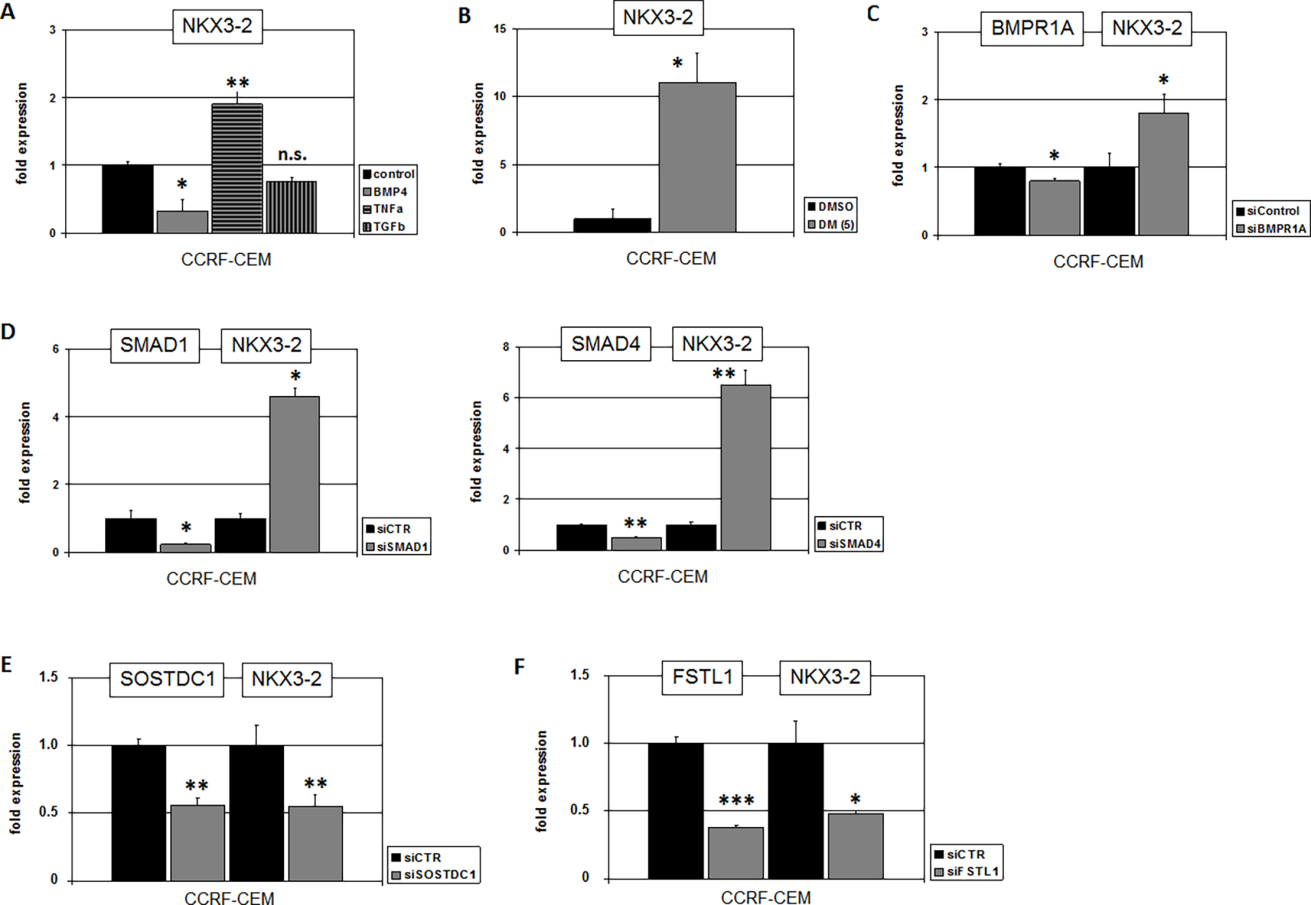
Regulation of NKX3-2 by the BMP-pathway. (A) RQ-PCR analysis of NKX3-2 in CCRF-CEM after treatment with BMP4, TNFa and TGFb demonstrates suppression by BMP-signalling and activation by TNFa-signalling. (B) RQ-PCR analysis of NKX3-2 in CCRF-CEM after treatment with BMP-receptor inhibitor DM supported gene suppression by BMP-pathway. (C) SiRNA-mediated knockdown of BMP-receptor 1A resulted in elevated expression of NKX3-2. (D) SiRNA-mediated knockdown of SMAD1 (left) and of SMAD4 (right) resulted in elevated expression of NKX3-2. SiRNA-mediated knockdown of SOSTDC1 (E) and of FSTL1 (F) resulted in reduced expression of NKX3-2.

To identify alternative deregulatory factors or pathways activating NKX3-2 in T-ALL we performed comparative expression profiling analysis of 79 T-ALL patient samples using dataset GSE42038. The 14 samples identified as positive for NKX3-2 were bioinformatically compared with the remaining 65 controls. This procedure revealed the top 250 most significant gene candidates differentially expressed. Among these genes we noticed overexpression of both NKX3-2 (p = 1.6x10^-17^) and SIX6 (p = 4.9x10^-5^) in addition to TNFRSF21 (p = 1.4x10^-4^), together with downregulation of BMP2K (p = 1.1x10^-4^), BMP10 (p = 4.7x10^-4^), and TGFBR1 (p = 1.1x10^-4^). These results suggested that NKX3-2 might be activated by TNFa- and suppressed by BMP- and/or TGFb-signalling. Subsequent treatment of CCRF-CEM with TNFa, BMP4 and TGFb confirmed transcriptional activation of NKX3-2 by TNFa-signalling and suppression by BMP-signalling while TGFb showed no significant effect (**Fig 4A**).

For a more detailed examination of the BMP-pathway in this context, we investigated several pathway components (ligands, receptors, regulators, nuclear mediators) by expression profiling in nine selected T-ALL cell lines as performed and shown previously [22]. These data showed downregulation of ligands and receptors in most cell lines but indicated overexpression of inhibitory regulators FSTL1 and SOSTDC1 in CCRF-CEM. Treatment of CCRF-CEM with BMP-receptor inhibitor dorsomorphin (DM) strongly enhanced NKX3-2 expression, supporting an inhibitory role for this pathway (**Fig 4B**). Moreover, siRNA-mediated knockdown of BMP-receptor BMPR1A (**Fig 4C**), and of the nuclear mediators SMAD1 and SMAD4 boosted NKX3-2 transcription (**Fig 4D**). Finally, knockdown of either FSTL1 or SOSTDC1 reduced NKX3-2 expression levels (**Fig 4E and 4F**). Thus, inhibition of the BMP-pathway via overexpression of particular negative regulators stimulated NKX3-2 expression in T-ALL.

**Fig 5:**
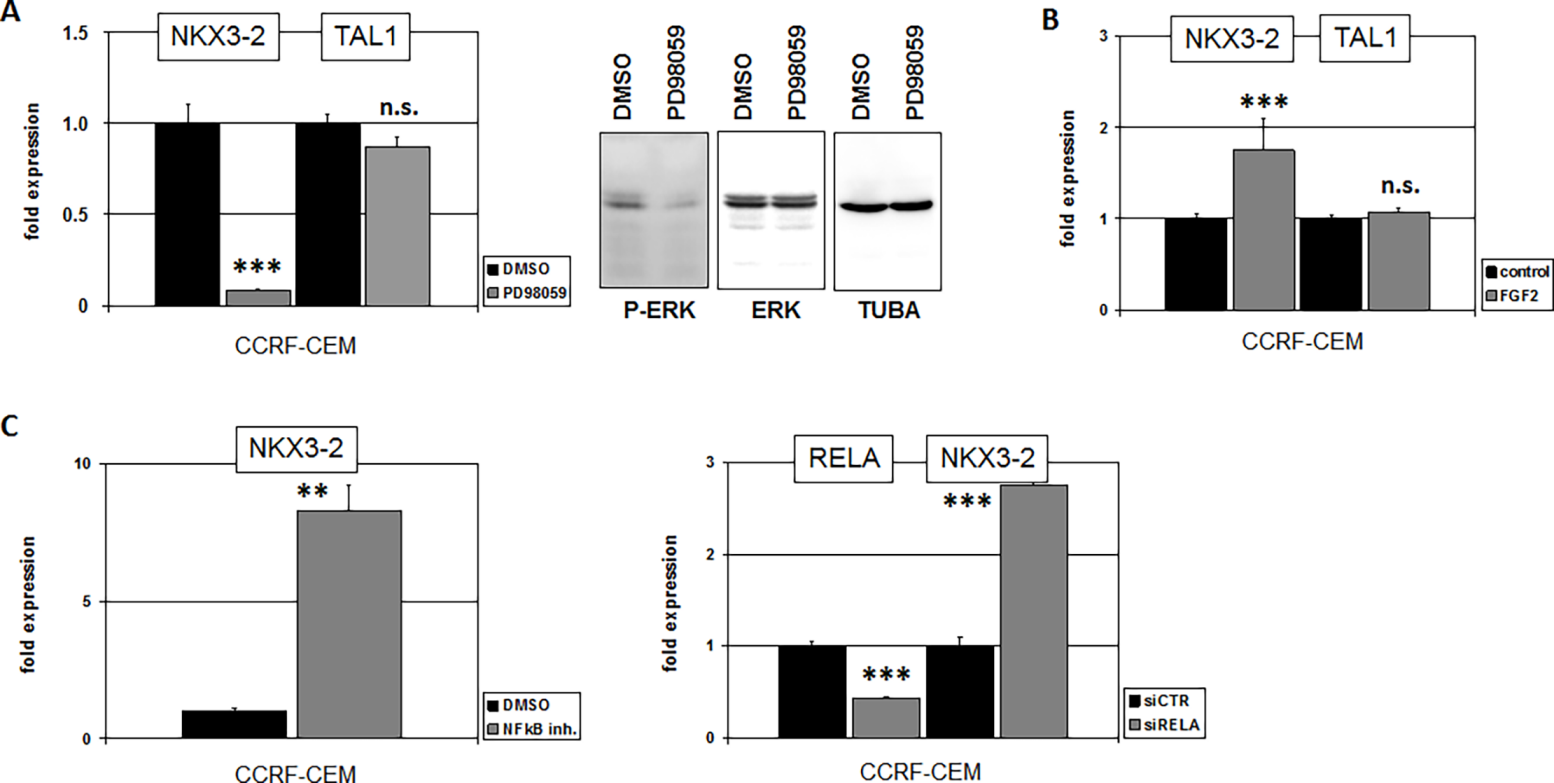
Regulation of NKX3-2 by MAPK-signalling. (A) RQ-PCR analysis of NKX3-2 in CCRF-CEM after treatment with MAPK-inhibitor PD98059 resulted in reduced NKX3-2 expression while control gene TAL1 showed no change (left). Western blot analysis of MAPK/ERK demonstrated reduced phosphorylation after treatment with PD98059 (right). (B) RQ-PCR analysis of CCRF-CEM after treatment with FGF2 resulted in elevated expression of NKX3-2 while TAL1 remained unchanged. (C) RQ-PCR analysis of NKX3-2 in CCRF-CEM after treatment with NFkB-inhibitor resulted in elevated gene expression (left). SiRNA-mediated knockdown of NFkB-factor RELA resulted in elevated expression of NKX3-2 (right).

TNF-signalling activates MAPK/ERK which performs multiple downstream functions [29,30]. To examine the impact of this pathway we treated CCRF-CEM with MAPK-inhibitor PD98059 which resulted in suppression of NKX3-2 expression while sparing control gene TAL1 (**Fig 5A**). FGF-signalling has also been described to activate MAPK [31]. Accordingly, treatment of CCRF-CEM with FGF2 activated NKX3-2 expression as strongly as TNFa while TAL1 again remained unperturbed (**Fig 5B**). NFkB is located downstream of both FGF2- and TNFa-signalling [31,32]. Treatment of CCRF-CEM with NFkB-inhibitor resulted in elevated NKX3-2 expression (**Fig 5C**), indicating an inhibitory impact of this TF. Consistently, siRNA-mediated knockdown of NFkB-factor RELA resulted in increased NKX3-2 transcription (**Fig 5C**). Taken together, we identified deregulated activities of BMP-, MAPK-, and NFkB-signalling pathways implicated in deregulation of NKX3-2 in T-ALL.

### Functional analyses of SIX6 and NKX3-2 in T-ALL cells

**Fig 6:**
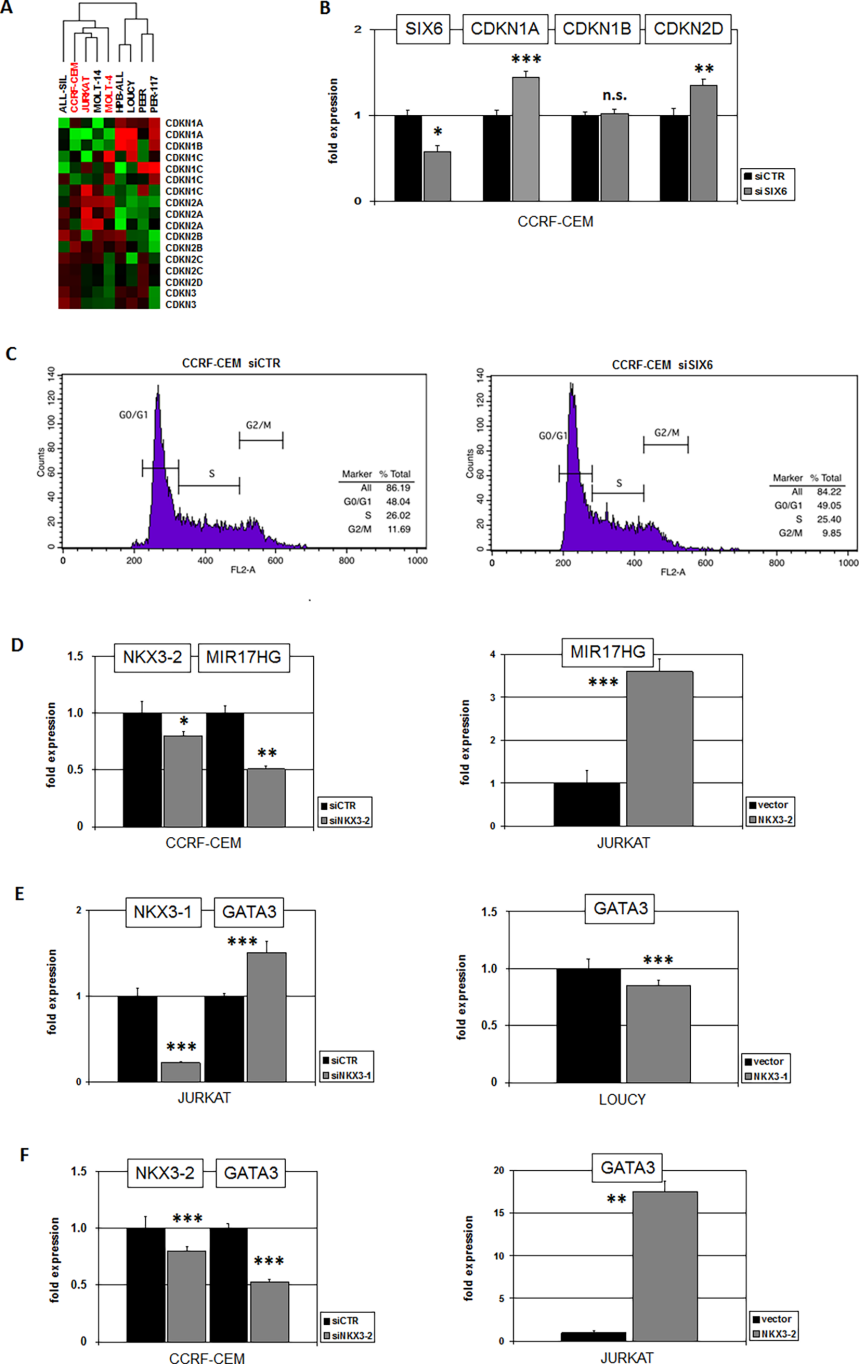
Functional analyses of SIX6 and NKX3-2. (A) Heatmap showing gene activities in 9 T-ALL cell lines of cyclin-dependent kinase inhibitors. Green represents low and red high expression levels. Cluster analysis showed clustering of SIX6-positive cell lines (red), indicating similarities in expression levels. (B) SiRNA-mediated knockdown of SIX6 in CCRF-CEM resulted in elevated expression of CDKN1A and CDKN2D while CDKN1B remained unchanged. (C) Cell cycle analysis of CCRF-CEM after siRNA-mediated knockdown of SIX6 by flow cytometry indicated no significant differences. (D) SiRNA-mediated knockdown of NKX3-2 in CCRF-CEM resulted in reduced expression (left), and forced expression of NKX3-2 in JURKAT in enhanced expression of MIR17HG (right). These results indicated that NKX3-2 activated MIR17HG expression in T-ALL. (E) SiRNA-mediated knockdown of NKX3-1 in JURKAT resulted in elevated expression (left), and forced expression of NKX3-1 in LOUCY in reduced expression of GATA3 (right). These results indicated that NKX3-1 inhibited GATA3 expression in T-ALL. (F) SiRNA-mediated knockdown of NKX3-2 in CCRF-CEM resulted in reduced expression (left), and forced expression of NKX3-2 in JURKAT in enhanced expression of GATA3 (right). These results indicated that NKX3-2 activated GATA3 expression in T-ALL.

Our expression profiling analysis supported the view that SIX6 represents a major target gene of NKL homeobox genes NKX3-2 and NKX3-1 in T-ALL. Physiologically, SIX6 activates cell cycle inhibitor CDKN2A in the developing eye, thus inhibiting cell proliferation in this embryonal tissue [33]. The results of gene expression cluster analysis for all cyclin-dependent kinase inhibitors (CDKI) in SIX6-positive and -negative T-ALL cell lines indicated a general regulatory relationship between SIX6 and CDKI expression in T-ALL (**Fig 6A**). To examine this potential connection in more detail we performed siRNA-mediated knockdown of SIX6 in CCRF-CEM and quantified transcripts of selected CDKIs by RQ-PCR. The results showed that SIX6 slightly but significantly inhibited the expression of CDKN1A and CDKN2D but not of CDKN1B (**Fig 6B**). However, cell cycle analysis performed after 48h by flow cytometry indicated no significant difference between SIX6-knockdown and control cells (**Fig 6C**), suggesting that homeobox gene SIX6 plays only a minor role in cell cycle regulation in T-ALL.

We have previously shown that NKL homeodomain proteins TLX1, TLX3 and NKX2-5 activate six micro-RNAs encoded by the host-gene MIR17HG [34]. To analyze a potential impact of NKX3-2 on this non-coding oncogene we performed siRNA-mediated knockdown of NKX3-2 in CCRF-CEM. This experiment showed concomitantly reduced expression of both genes, indicating that NKX3-2 also activated transcription of MIR17HG (**Fig 6D**). Furthermore, forced expression of NKX3-2 in JURKAT cells resulted in enhanced MIR17HG expression supporting an activatory impact (**Fig 6D**). Thus, while NKX3-2 in addition to other oncogenic NKL homeodomain TFs activates MIR17HG, NKX3-1 suppresses this gene as described previously [23].

GATA3 encodes a Zn-finger TF which plays a basic role in the development of T-cells and prostate. In both cell/tissue types GATA3 mediates transcriptional activation of NKL homeobox gene NKX3-1 [23,35]. To analyze if NKX3-1 and/or NKX3-2 regulate the expression of GATA3 we performed knockdown and overexpression experiments in T-ALL cell lines. The results indicated that while NKX3-1 inhibited GATA3 (**Fig 6E**), NKX3-2 activated its expression (**Fig 6F**). Thus, the target genes MIR17HG and GATA3 were differentially regulated by these closely related NKL homeodomain TFs.

### NKX3-2 and FOXG1 are transcriptional activators of NKX2-5

**Fig 7:**
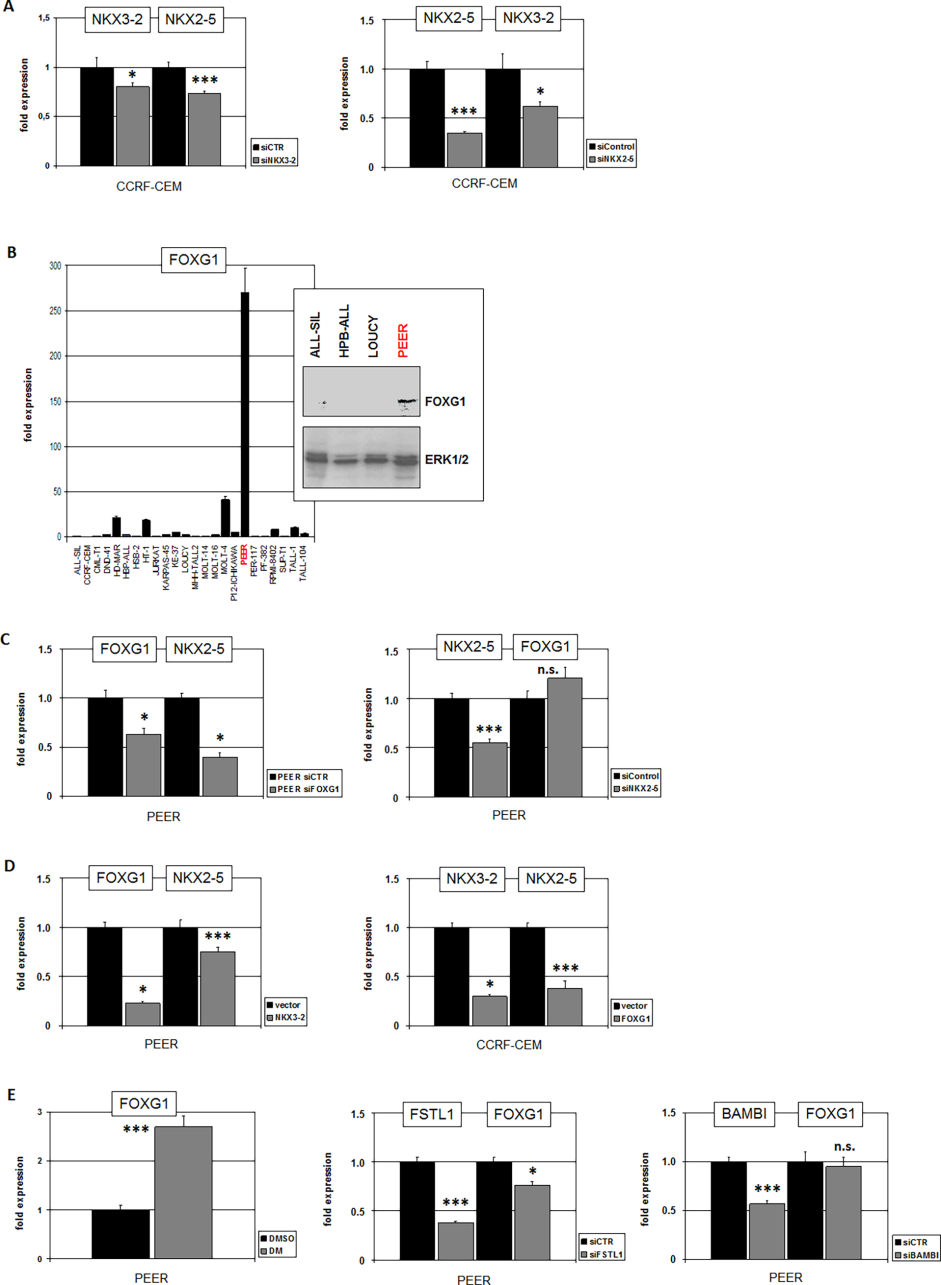
NKX3-2 and FOXG1 are activators of NKX2-5 in T-ALL. (A) SiRNA-mediated knockdown in CCRF-CEM of NKX3-2 (left) and of NKX2-5 (right) resulted in concomitantly reduced expression of NKX2-5 and NKX3-2, respectively. These results indicated mutually activation of NKX3-2 and NKX2-5. (B) RQ-PCR analysis of FOXG1 in 24 T-ALL cell lines demonstrated enhanced expression levels in PEER. Western blot analysis of FOXG1 in 4 T-ALL cell lines confirmed FOXG1 protein expression in PEER. ERK served as loading control (insert). (C) SiRNA-mediated knockdown of FOXG1 in PEER (left) resulted in concomitantly reduced expression of NKX2-5 indicating that FOXG1 activates NKX2-5 transcription. SiRNA-mediated knockdown of NKX2-5 (right) showed no change in expression levels of NKX2-5 discounting mutual regulation. (D) Forced expression of NKX3-2 in PEER resulted in reduced expression of FOXG1 and NKX2-5 (left). Forced expression of FOXG1 in CCRF-CEM resulted in reduced expression of NKX3-2 and NKX2-5. (E) Treatment of PEER with BMP-pathway inhibitor DM resulted in elevated expression levels of FOXG1 (left), indicating a suppressive impact of this pathway on FOXG1. SiRNA-mediated knockdown of FSTL1 in PEER (middle) resulted in concomitantly reduced expression of FOXG1 while knockdown of BAMBI showed no effect on FOXG1 expression (right).

The expression data for NKX3-2 and NKX2-5 showed combined activities in both spleen tissue and T-ALL cell line CCRF-CEM (**Fig 2D**), suggesting co-regulation. To test this potential relationship we performed siRNA-mediated knockdown of NKX3-2 and NKX2-5 in CCRF-CEM and quantified their transcript levels (**Fig 7A**). This analysis demonstrated mutual activation of these homeobox genes which may reflect physiological conditions in spleen development.

To examine if the NKX2-5 positive but NKX3-2 negative T-ALL cell line PEER harbors an alternative factor which contributes to NKX2-5 activation we performed comparative expression profiling of PEER and CCRF-CEM. We thus compiled a shortlist of candidates, containing 4717 genes which differed in their activity of at least 4-fold (data not shown). In PEER this approach revealed overexpression of the forkhead-box gene FOXG1 which encodes a developmental TF coexpressed with NKX2-5 in the developing thymus of mice [36]. Quantification of FOXG1 transcripts by RQ-PCR and analysis of FOXG1 protein by westernblot in T-ALL cell lines confirmed exclusively high expression levels in PEER (**Fig 7B**). SiRNA-mediated knockdown of FOXG1 and NKX2-5 in PEER demonstrated that FOXG1 activated NKX2-5 while no reciprocal action was detectable (**Fig 7C**). Interestingly, forced expression of FOXG1 in CCRF-CEM and of NKX3-2 in PEER resulted in decreased expression of NKX3-2 and FOXG1, respectively, in addition to decreased expression levels of NKX2-5 in both cell lines (**Fig 7D**). Thus, NKX3-2 and FOXG1 represent mutually inhibitory activators of NKX2-5.

We have shown that the BMP-pathway suppressed the expression of NKX2-5 activator NKX3-2 in CCRF-CEM. Accordingly, aberrant overexpression of two BMP-pathway inhibitors was detected in this cell line by gene expression profiling. The same approach indicated overexpression of BMP-inhibitors FSTL1 and BAMBI in PEER [22]. Therefore, we investigated the impact of the BMP-pathway on FOXG1 by treatment of PEER with BMP-receptor inhibitor DM. This experiment showed that BMP-signalling suppressed FOXG1 expression in PEER. We then tested the influence of the activated BMP-pathway inhibitors on FOXG1 expression via siRNA-mediated knockdown (**Fig 7E**). The results indicated that FSTL1 but not BAMBI contributed to the activation of FOXG1 in PEER. Collectively, our data show that cytogenetic activation of NKX2-5 which we reported previously is respectively boosted by splenic and thymic TFs, NKX3-2 and FOXG1, both of which are subject to BMP-pathway regulation.

## Discussion

**Fig 8:**
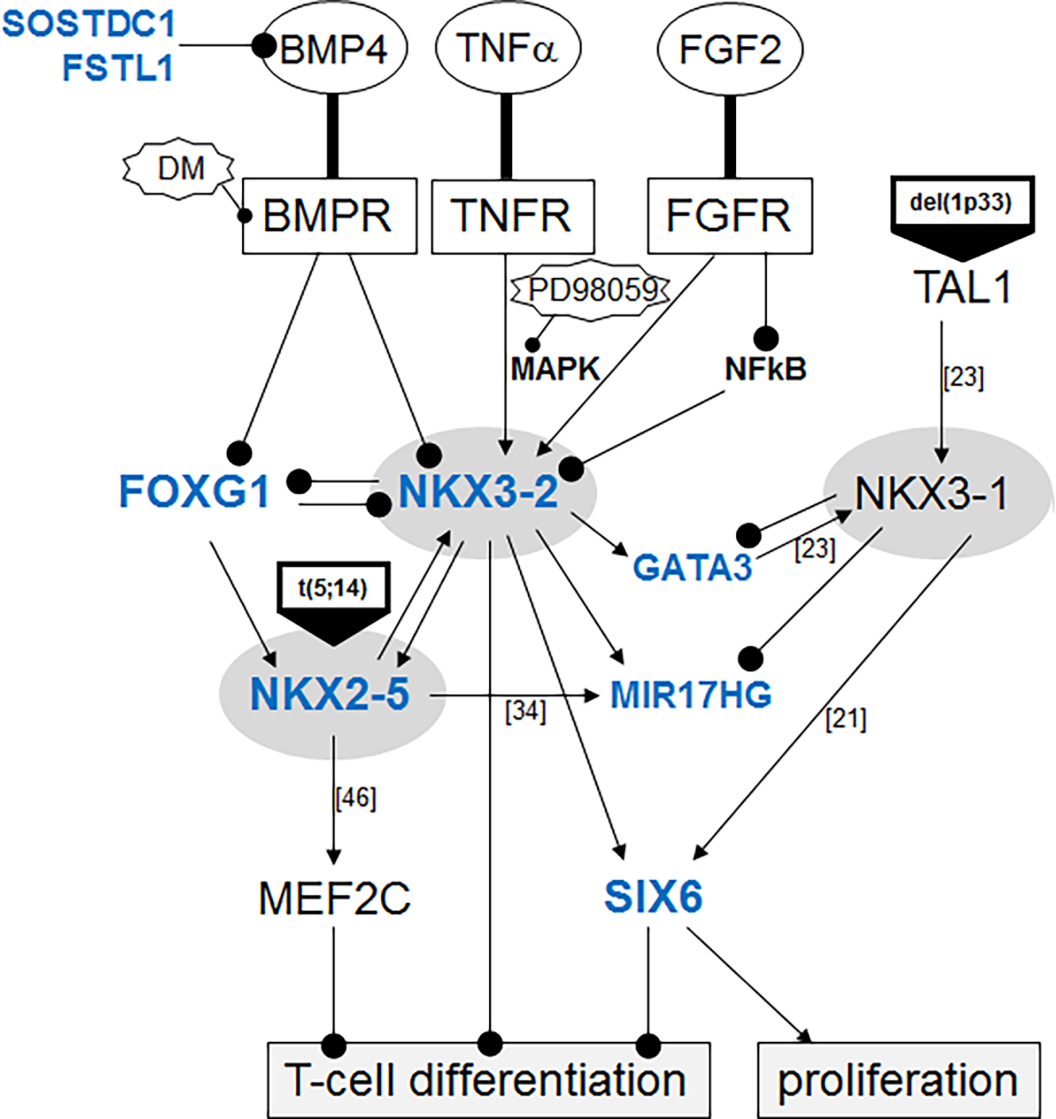
Aberrant gene regulatory network with NKX3-2. This diagram summarizes the results of this study (genes in bold letters) and of previous publications (indicated by the number of the according citation). Collectively, this aberrant gene network essentially contributes to blocked T-cell differentiation because all implicated genes encode basic developmental regulators.

Here, we described the identification of a novel deregulated NKL homeobox gene, NKX3-2, in T-ALL. We mined public expression profiling data of patients and used the NKX3-2 positive cell line CCRF-CEM as a model to analyze mechanisms of aberrant activation and downstream targets. A summary of the results from this study in addition to published data is shown in **Fig 8**. While excluding chromosomal rearrangements, we demonstrated that NKX3-2 was activated via MAPK-signalling and suppression of the inhibitory BMP-pathway. NKX3-2 and NKX3-1 activated the expression of homeobox gene SIX6, and differentially regulated MIR17HG and GATA3. Furthermore, NKX3-2 and NKX2-5 proved mutually stimulatory while in contrast NKX3-2 and FOXG1 operated as mutual inhibitors. FOXG1 was shown to be an alternative regulator of NKX2-5 exclusively expressed in PEER cells. All homeobox genes analyzed are developmental regulators, indicating that their aberrant activity upsets differentiation of T-cell progenitors.

NKL homeobox gene NKX3-2 is closely related to NKX3-1 displaying, nevertheless, several differences in their sequences which may give rise to functional disparities [10]. However, both proteins show nearly identical DNA binding site characteristics [37]. While NKX3-1 is expressed in hematopoietic stem cells and early double-negative T-cell progenitors, NKX3-2 remains silent during hematopoiesis and is involved in the embryonal development of the spleen [17,27]. Betraying their importance as malignant activators in pediatric T-ALL patients, we observed similarly raised frequencies of aberrant NKX3-2 (~18%) and TLX3 (~22%) but somewhat lower patient counts affecting TLX1 (7%), demonstrating a frequent involvement of this subclass member in this malignancy in children.

Aberrant activation of both, NKX3-2 and NKX3-1, avoids chromosomal rearrangement [21]. While NKX3-1 is regulated by the oncogenic TAL1-complex [23], NKX3-2 activation occurred via deregulated signalling-pathways including MAPK and BMP. The MAPK-pathway is activated by several ligands including TNFa and FGF2 [31,32]. Their signalling is physiologically involved in T-cell differentiation implying that deregulation of MAPK-activity mediates transformation of T-cell progenitors [29,30]. The BMP-pathway is also involved in T-cell development, while its aberrant suppression in T-ALL promotes activation of NKL homeobox genes NKX3-2 as shown here or MSX1 as shown previously [22,38]. Overexpression of BMP-pathway inhibitor FSTL1 resulted in NKX3-2 deregulation while overexpression of inhibitor CHRDL1 contributed to MSX1 activation [22]. Thus, active BMP-signalling is fundamental for normal T-cell differentiation while its suppression mediates aberrant expression of particular oncogenic NKL homeodomain factors. Our data indicated a suppressive role of NFkB-signalling on NKX3-2 expression. This relationship may represent a physiological function of NFkB to prevent activation of NKX3-2 in T-cell development. Interestingly, NKX3-2 has been shown to interact with NFkB-factor RELA [39], demonstrating a mutual regulation between this NKL homeodomain protein and NFkB-signalling. Furthermore, this protein-protein interaction may underlie our observation that NKX3-2 protein is located in the cytoplasma. SMAD1 and SMAD4 are additional TFs which are able to interact with NKX3-2 protein [40]. Thus, NKX3-2 may retain suppressing TFs in the cytoplasma, enhancing transcriptional activation of its own gene.

The embryonal development of the spleen is regulated by a group of homeobox genes including NKL subclass members NKX2-5, TLX1 and NKX3-2 which form a regulatory network [28]. These three genes are not expressed in normal hematopoietic cells but aberrantly activated in T-ALL subsets [17]. Our data show that NKX3-2 activated NKX2-5 in T-ALL cell line CCRF-CEM. However, whether this regulatory connection also plays a role in the spleen remains unknown. In T-ALL cell line PEER we identified another transcriptional activator of NKX2-5, namely FOXG1. FOXG1 is not expressed in the developing spleen but in the developing thymus [36]. This was striking because NKX2-5 is expressed during murine embryogenesis in both spleen and thymus [14,41]. Thus, we identified two different aberrant activators of NKX2-5 in T-ALL which may recapitulate embryonal activities operating in developing lymphatic organs. Interestingly, FOXN1 belongs together with FOXG1 to the FOX-family of TFs and represents a master gene for thymopoiesis [42]. Furthermore, the related FOX-factors FOXN2 and FOXN3 constitute two tumor suppressor genes in T-ALL [43], again highlighting a relationship between related TFs and the development of T-cells and lymphatic organs. Thus, aberrant activation of splenic/thymic developmental pathways may represent a more widespread feature of oncogene deregulation in T-ALL. Moreover, our results may suggest that other translocated oncogenes require particular TFs to support their activity as well.

SIX6 is a target gene of both NKX3-1 and NKX3-2 and shows aberrant activation in T-ALL subsets [12,21]. Physiological expression of SIX6 has been described in eye development [24]. In this context SIX6 is regulated via defined enhancers and TFs neither of which are known to involve NKX3-1 or NKX3-2 [44,45]. During eye-development SIX6 sets the balance between proliferation and differentiation in the retina and suppresses specific non-lineage genes [33,46]. Suppression of non-lineage genes has also been described for NKL homeodomain factors, including NKX3-1 in developing prostate, indicating that this feature may represent a general effect of these oncogenes in T-ALL [47]. In contrast, NKX2-5 activates the developmental regulator MEF2C which is involved in the differentiation of cardiomyocytes and B-cells but not of T-cells and represents a strong oncogene in T-ALL [48,49]. MIR17HG encodes six different micro-RNAs and is activated in T-ALL by several NKL homeodomain proteins including NKX3-2 as described here [34]. In contrast, NKX3-1 inhibits the expression of MIR17HG in this malignancy [23]. GATA3 represents a novel target gene of these two NKL homeodomain proteins in T-ALL. Similar to MIR17HG, NKX3-1 inhibited GATA3 expression while NKX3-2 operates as an activator demonstrating additional gene regulatory differences between these closely related TFs. In T-cell lymphoma GATA3 and MIR17HG are overexpressed while in early T-cell progenitor ALL these genes are inactivated or downregulated [50–54]. Thus, GATA3 and MIR17HG operate in particular T-cell malignancies as oncogenes or tumor suppressors. This finding might indicate pathological differences between NKX3-1 and NKX3-2 positive T-ALL cases.

Disturbing differentiation in T-cell progenitors has been shown for TLX1 by identification of a particular deregulated gene network and its potential to interact with the TF ETS1, thereby suppressing rearrangement and expression of the T-cell receptor genes [55,56]. NKX3-1 binds a related ETS-factor in the prostate highlighting the importance of this type of protein interaction in cell differentiation processes [57]. Moreover, all NKL proteins possess an EH1-domain which mediates interaction with corepressors of the TLE-family [9]. This interaction presumably plays a basic role in T-ALL [58,59]. Of note, SIX6 interacts with TLE-repressors as well [60], supporting the idea that this function may represent a common pathological mechanism of onco-proteins in this malignancy.

Master genes have a fundamental impact in cell and tissue differentiation and are able to perform reprogramming and transdifferentiation in a transgene context. While bHLH factor MYOD represents a master gene which can alone convert fibroblasts into muscle cells [61], usually small groups of master genes are necessary for transdifferentiations of this cell type. Often, these groups contain NKL homeobox genes like NKX2-5 to generate cardiomyocytes or NKX3-1 to generate prostate tissue [62,63]. This interdependence on particular cofactors may explain that aberrant expression of individual NKL homeobox genes in T-cell progenitors results in differentiation arrest and not in conversions into other non-hematopoietic cell types.

Expression analysis of all 48 human NKL homeobox genes in early hematopoiesis, lymphopoiesis and T-cell progenitors highlighted nine genes, including NKX3-1 and MSX1, which constitute an NKL-code [17]. Some of these genes show reciprocal regulation, generating a network in stem/progenitor cells. While all NKL homeobox homeobox genes are silenced in mature T-cells, MSX1 remained active in NK-cells [64]. Accordingly, MSX1 operates as a tumor suppressor in NK-cells and as an oncogene in T-cell progenitors [22,64]. Thus, developing T-cells suppress their NKL-activity to terminate differentiation and appear, therefore, exceedingly susceptible for this type of oncogenes which disturb this process. NKX3-2 represents a novel oncogenic member of this subclass. The data of this study describing its deregulation and function may contribute to understand the pathological role of this important type of oncogenes in T-ALL.
